# Supplementary material for: Probabilistic reconstruction of measles transmission clusters from routinely collected surveillance data
Source: J R Soc Interface. 2020 Jul 1;17(168):20200084. doi: 10.1098/rsif.2020.0084 (PMC7423430; doi:10.1098/rsif.2020.0084)
Supplement: Additional figures [file rsif20200084supp1.docx]

SUPPLEMENT: Probabilistic reconstruction of measles transmission clusters from routinely collected surveillance data.

Authors:

Alexis Robert^1,2^, Adam J. Kucharski^1,2^, Paul A. Gastañaduy^3^, Prabasaj Paul^4^, Sebastian Funk^1,2^

# Authors’ affiliations:

1. Centre for the Mathematical Modelling of Infectious Diseases, London School of Hygiene & Tropical Medicine, Keppel Street, London, UK

2. Department of Infectious Disease Epidemiology, London School of Hygiene & Tropical Medicine, Keppel Street, London, UK

3. Division of Viral Diseases, Centers for Disease Control and Prevention, Atlanta, Georgia

4. Division of Healthcare Quality Promotion, Centers for Disease Control and Prevention, Atlanta, Georgia

# Description of the US dataset

**
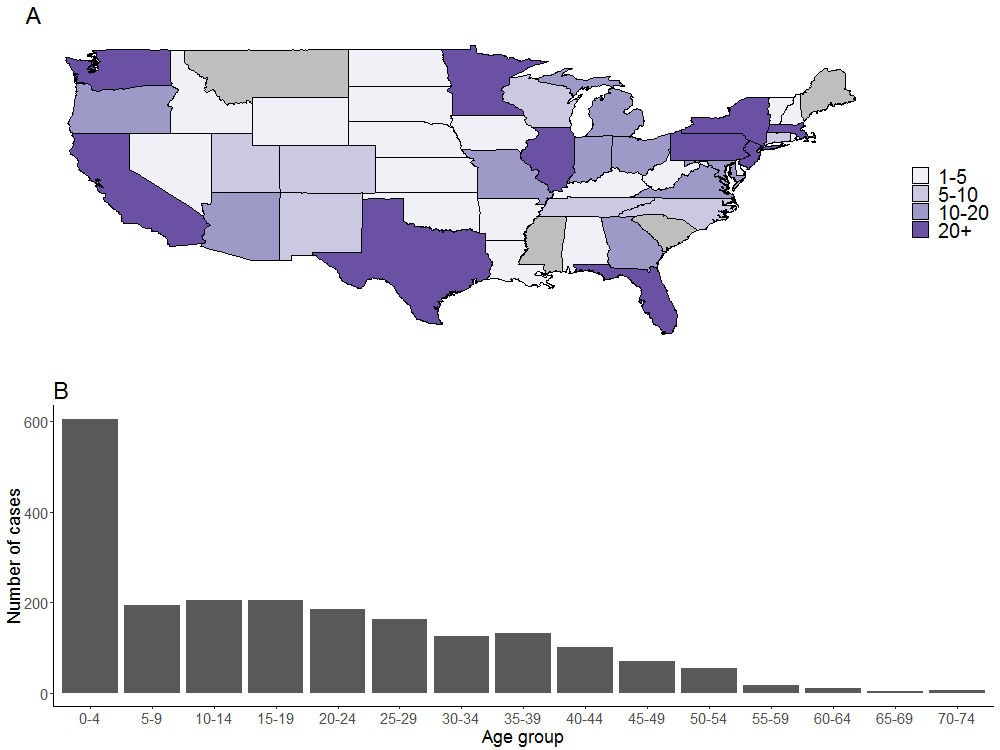
**

Figure S1: Description of the cases and clusters reported in the United States between 2001 and 2016. Panel A: number of clusters reported per state in the contact tracing investigations (5 clusters cover several states) and Panel B: Age distribution of the cases. Data from CDC’s National Notifiable Disease Surveillance System. Hawaii and Alaska and not shown in Panel A.

# Evaluation cluster matching


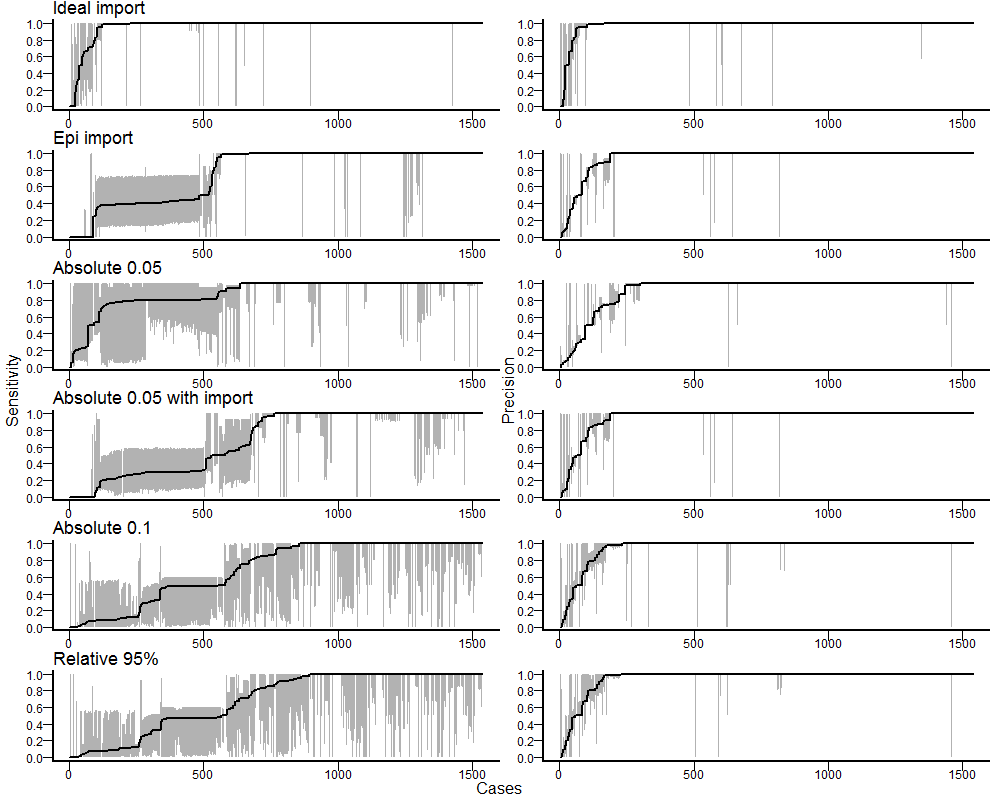


Figure S2: Confidence intervals of the sensitivity (right panels) and precision (left panels) of the clusters of each case in the different runs. For each case, the sensitivity is the proportion of cases from the reference cluster that were correctly inferred, the precision is the proportion of cases from the inferred cluster that were part of reference cluster. Grey areas represent the 95% credibility intervals, and the black line represents the median values of sensitivity or precision across all iterations.

# Posterior distribution and convergence


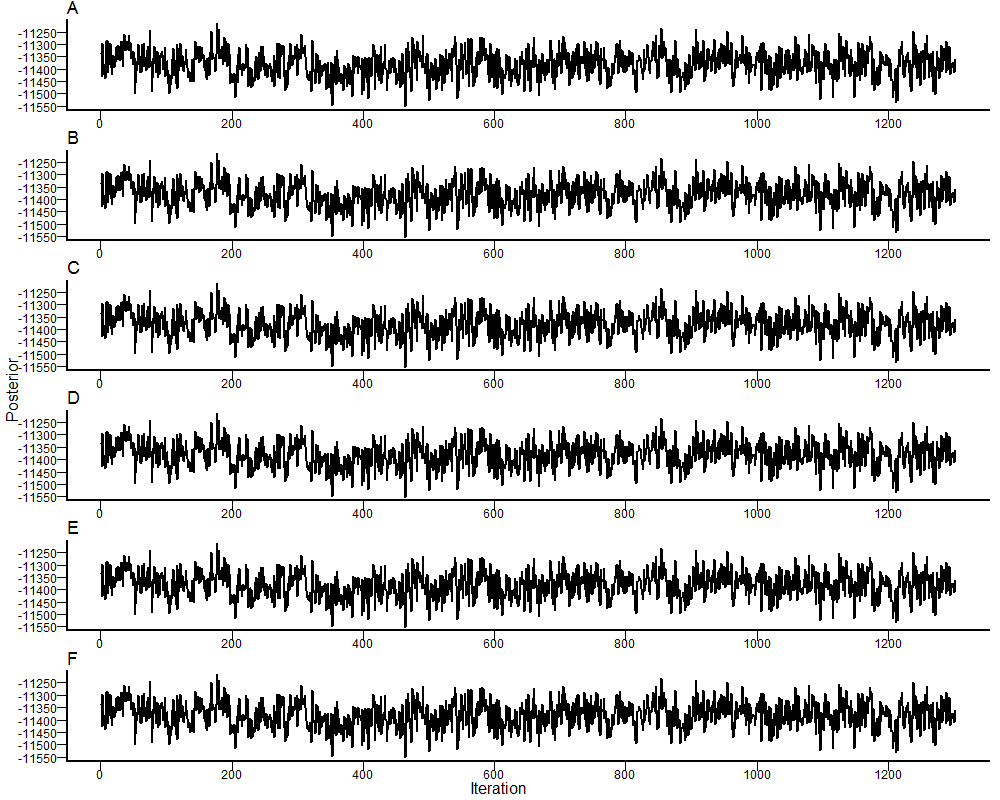


Figure S3: Trace of the posterior value of the MCMC runs after removing the burnin section and thinning. A: Ideal imports; B: Epi imports; C: Absolute threshold, $k=0.05$ D: Absolute threshold, $k=0.05$ with prior information on imports; E: Absolute threshold, $k=0.1$; F: Relative threshold upper $\lambda=97.5\%$quantile.

# Clusters stratified by state

## Scenario 1


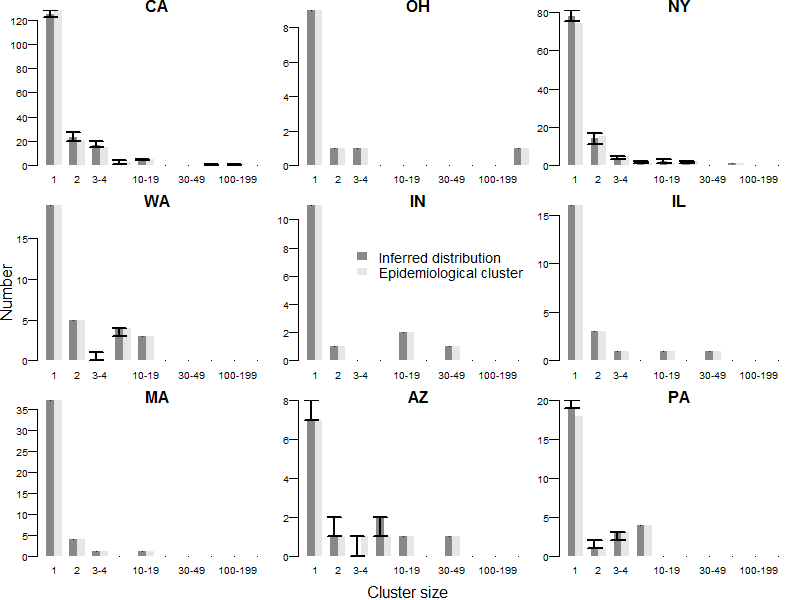


Figure S4: Comparison of the inferred and reference cluster size distributions in the nine states with most cases declared between 2001 and 2016 in scenario 1. Arrows represent the 95% credibility intervals of each estimate.

## Scenario 2


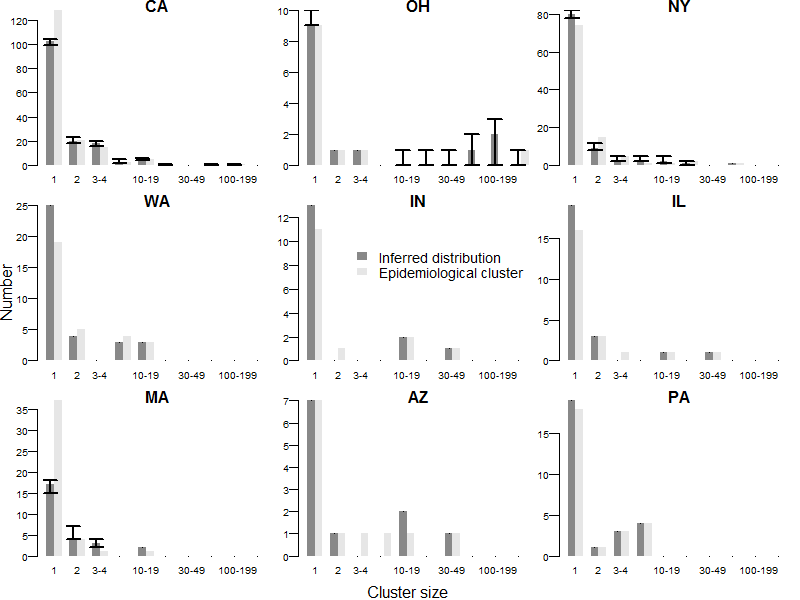


Figure S5: Comparison of the inferred and reference cluster size distributions in the nine states with most cases declared between 2001 and 2016 in scenario 2. Arrows represent the 95% credibility intervals of each estimate.

## Scenario 3

### Absolute threshold $\boldsymbol{k=0.05}$


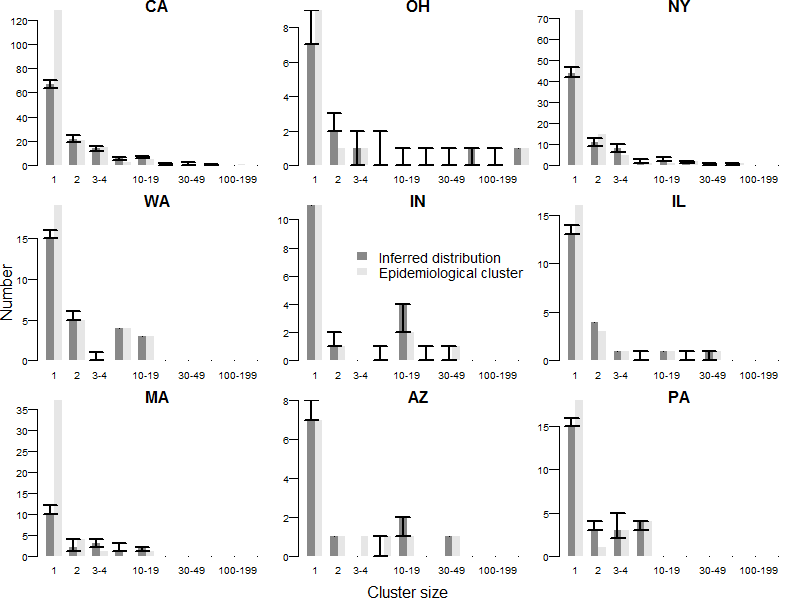


Figure S6: Comparison of the inferred and reference cluster size distributions in the nine states with most cases declared between 2001 and 2016 in scenario 3, with an absolute threshold $k=0.05$ and no prior information on the import status of cases. Arrows represent the 95% credibility intervals of each estimate.

### Relative threshold $\boldsymbol{k=95\%}$


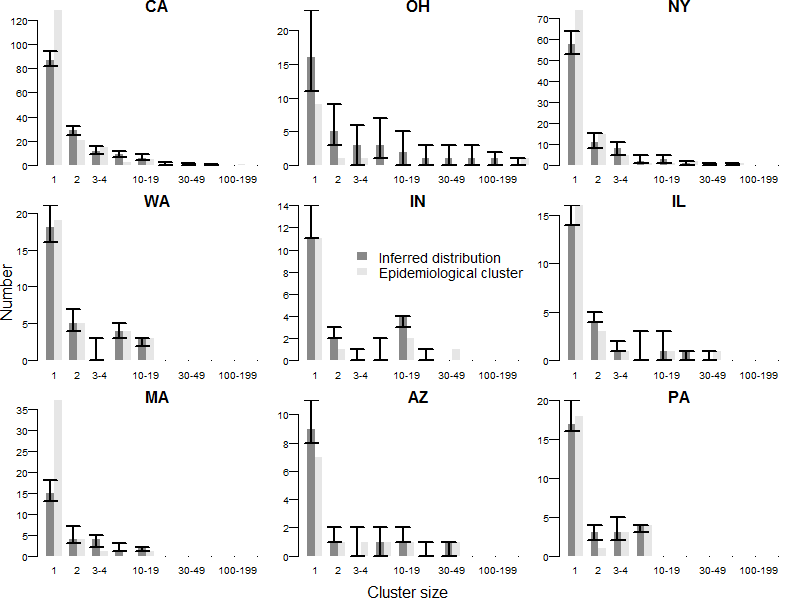


Figure S7: Comparison of the inferred and reference cluster size distributions in the nine states with most cases declared between 2001 and 2016 in scenario 3, with a relative threshold$k=95\%$ and no prior information on the import status of cases. Arrows represent the 95% credibility intervals of each estimate.

### Epidemiological imports, and absolute threshold $\boldsymbol{k=0.05}$


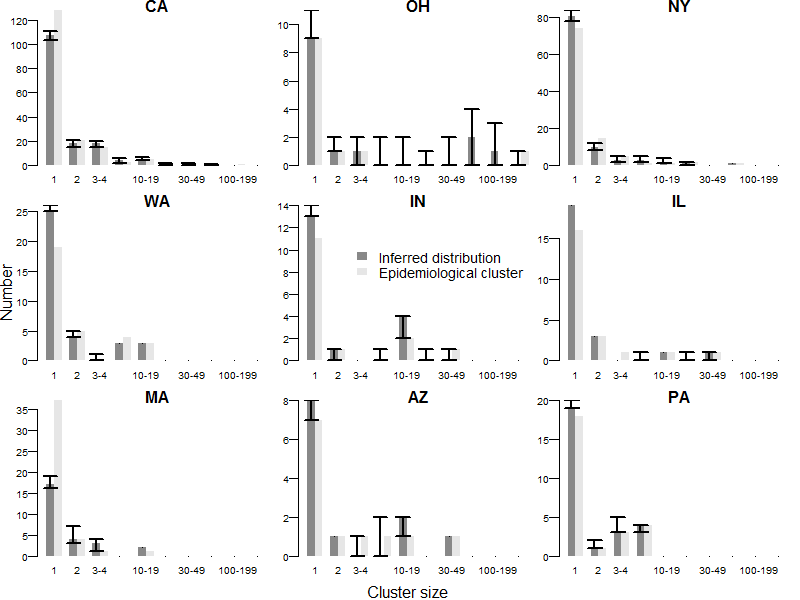


Figure S8: Comparison of the inferred and reference cluster size distributions in the nine states with most cases declared between 2001 and 2016 in scenario 3, with an absolute threshold $k=0.05$ and using the import status distribution from the contact tracing investigations as prior information. Arrows represent the 95% credibility intervals of each estimate.

# Parameter estimates


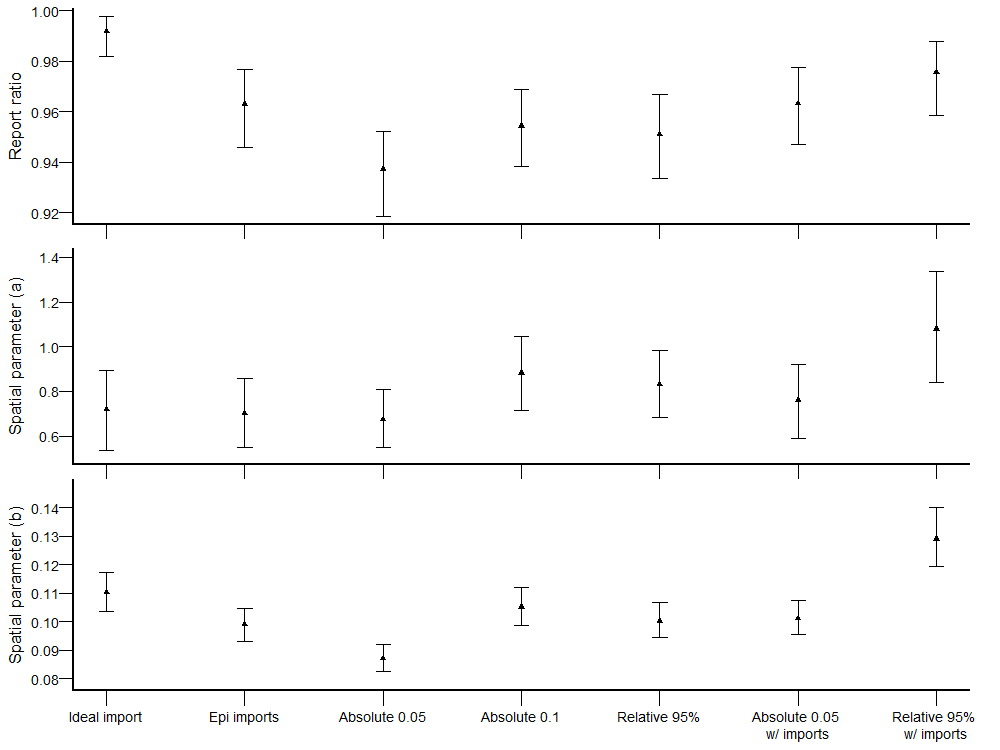


Figure S9: Estimation of A) the report ratio$\rho$, B) the spatial parameter $a$ and C) the spatial parameter $b$ in each scenario. The dots represent the median estimate, and the arrows correspond to the 95% credibility interval. The estimates were obtained after burnin and thinning.

# Distance between transmission


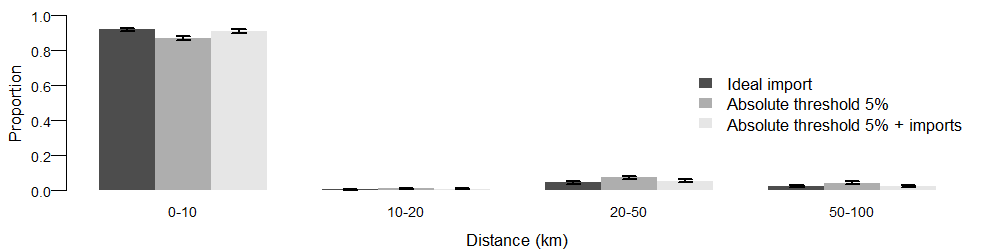


Figure S10: Distribution of the distance between connected cases in scenario 1, scenario 3 without prior and scenario 3 with prior information. Arrows correspond to the 95% credibility interval.

# Impact of different components of likelihood


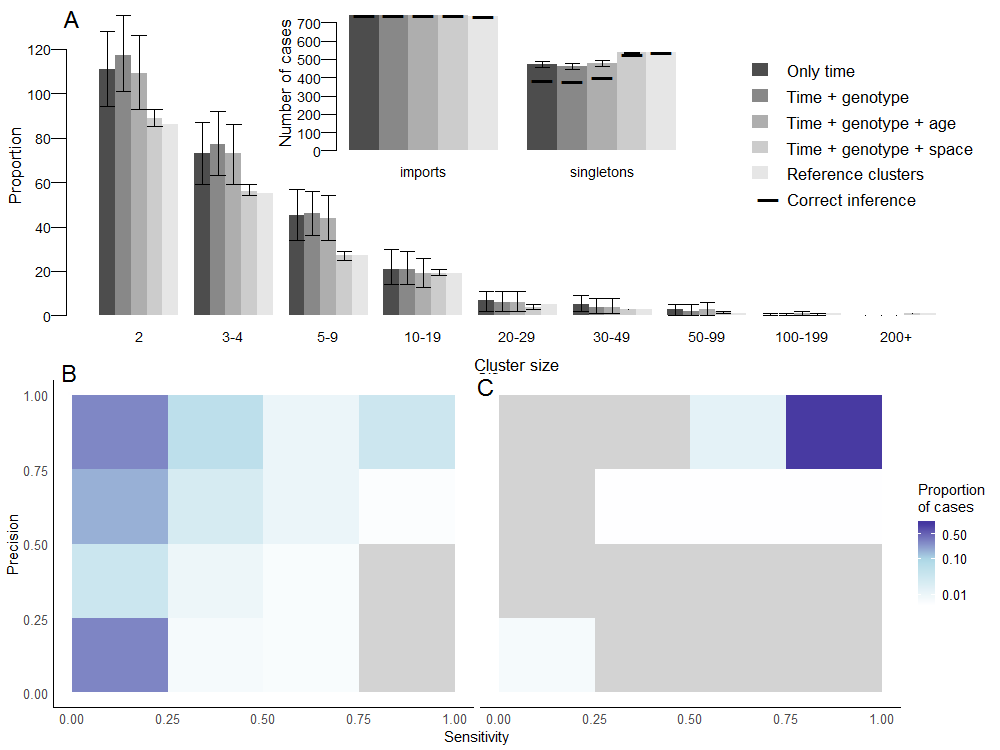


Figure S11: Description of transmission clusters inferred excluding certain components of the likelihood. In this example, the import status of import was not inferred, and taken from the ancestor in each cluster (Scenario 1). Panel A: Cluster size distribution using 1) only the time component of likelihood; 2) time and genotype; 3) time genotype and age; 4) time, genotype and space; compared to the reference clusters (lighgrey). Arrows represent the 95% credibility intervals of each estimate. Only clusters containing at least 2 cases are represented. Inset: Number of imports and number of isolated cases (singletons). For each scenario, the horizontal dark line represents the number of imports that are also imports in the reference clusters, same for singletons. Panel B: Heatmap representing the precision and sensitivity of the clusters for each case when only time and genotype are used to infer the transmission clusters, the cases were classified in a category depending on the proportion of their reference cluster that were inferred in the same cluster (x-axis) and the proportion of mismatches in the inferred cluster. Panel C: Same for time, genotype and space.

# Number of secondary transmissions, overall and per state

## Overall


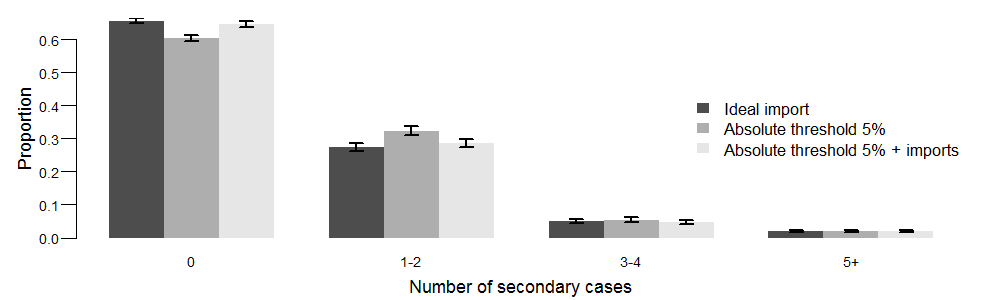


Figure S12: Distribution of the number of secondary cases caused by each case in scenario 1, scenario 3 without prior and scenario 3 with prior information. Arrows correspond to the 95% credibility interval. Cases were classified in groups of transmitter (no further transmission; 1-2 subsequent cases; 3-4 subsequent cases and more than 5 subsequent cases).

## Maps per state


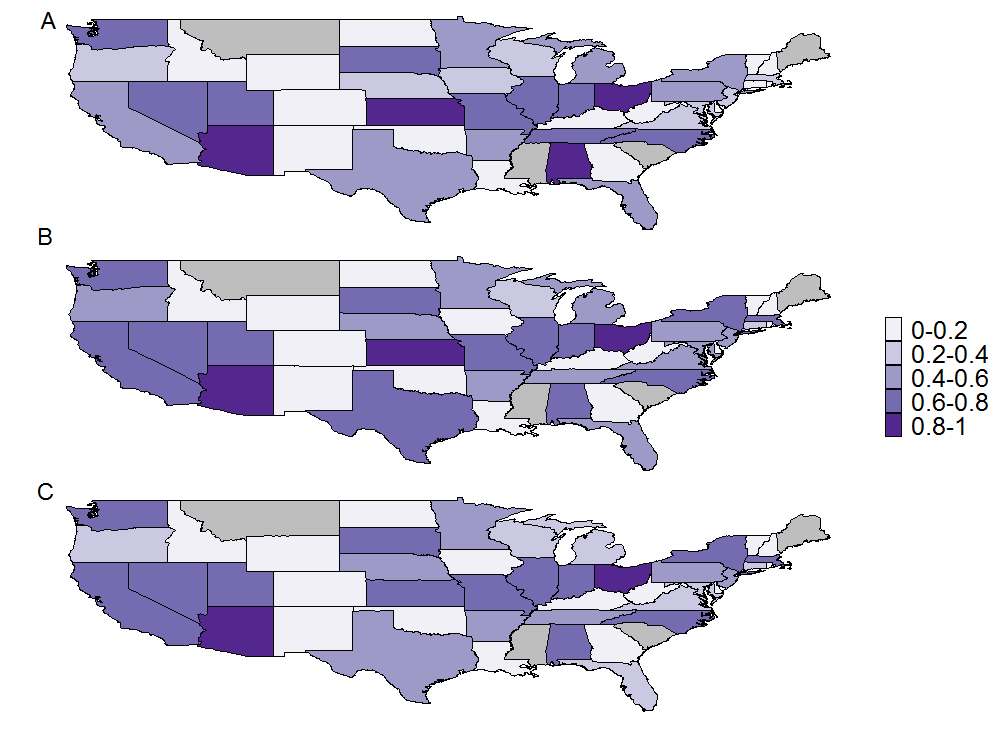


Figure S13: Average number of secondary cases caused by each case stratified by state in A) scenario 1, B) scenario 3 without prior and C) scenario 3 with prior information.

# Impact of the proportion of genotype reported. Inference on simulated data


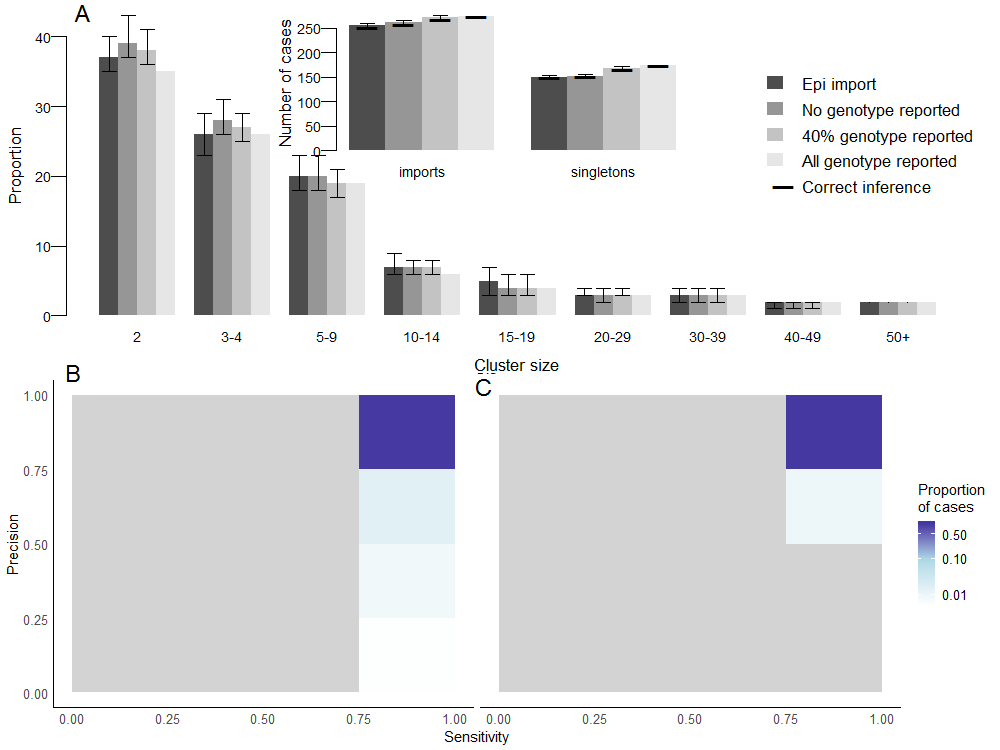


Figure S14: Description of transmission clusters inferred using simulated data (toy_outbreak_long in the o2geosocial package), depending on the proportion of genotyped cases in the data. Panel A: Cluster size distribution when 1) none of the cases was genotyped, 2) 40% of the cases were genotyped (similar to the US dataset) 3) All the cases were genotyped; compared to the reference clusters (lighgrey). Arrows represent the 95% credibility intervals of each estimate. Only clusters containing at least 2 cases are represented. Inset: Number of imports and number of isolated cases (singletons). For each scenario, the horizontal dark line represents the number of imports that are also imports in the reference clusters, same for singletons. Panel B: Heatmap representing the precision and sensitivity of the clusters for each case when no genotype was reported are used to infer the transmission clusters, the cases were classified in a category depending on the proportion of their reference cluster that were inferred in the same cluster (x-axis) and the proportion of mismatches in the inferred cluster. Panel C: Same when all cases were genotyped.
